# Field evaluation of a new antibody-based diagnostic for *Schistosoma haematobium* and *S. mansoni* at the point-of-care in northeast Zimbabwe

**DOI:** 10.1186/1471-2334-14-165

**Published:** 2014-03-26

**Authors:** Norman Nausch, Emily M Dawson, Nicholas Midzi, Takafira Mduluza, Francisca Mutapi, Michael J Doenhoff

**Affiliations:** 1 Institute of Immunology and Infection Research, Centre for Immunity, Infection and Evolution, Ashworth Laboratories, West Mains Road, School of Biological Sciences, University of Edinburgh, Edinburgh EH9 3JT, UK; 2 School of Life Sciences, University of Nottingham, University Park, Nottingham NG7 2RD, UK; 3 National Institutes of Health Research, Box CY 573 Causeway, Harare, Zimbabwe; 4 Department of Biochemistry, University of Zimbabwe, P.O. Box 167Mount Pleasant, Harare, Zimbabwe; 5 Current Address: Pediatric Pneumology and Infectious Diseases Group, Department of General Pediatrics, Neonatology, and Pediatric Cardiology, University Children’s Hospital, 40225 Duesseldorf, Germany; 6 Current Address: Department of Medical Microbiology, University of Zimbabwe, P.O. Box A178 Avondale, Harare, Zimbabwe

**Keywords:** Schistosomiasis, Diagnosis, Antibody-detection, Point-of-care, Neglected tropical disease

## Abstract

**Background:**

Rapid diagnostic tests (RDTs) for use at the point-of-care (POC) are likely to become increasingly useful as large-scale control programmes for schistosomiasis get underway. Given the low sensitivity of the reference standard egg count methods in detecting light infections, more sensitive tests will be required to monitor efforts aimed at eliminating schistosomiasis as advocated by the World Health Assembly Resolution 65.21 passed in 2012.

**Methods:**

A recently developed RDT incorporating *Schistosoma mansoni* cercarial transformation fluid (SmCTF) for detection of anti-schistosome antibodies in human blood was here evaluated in children (mean age: 7.65 years; age range: 1-12 years) carrying light *S. mansoni* and *S. haematobium* infections in a schistosome-endemic area of Zimbabwe by comparison to standard parasitological techniques (i.e. the Kato-Katz faecal smear and urine filtration). Enzyme-linked immunosorbent assays (ELISAs) incorporating *S. haematobium* antigen preparations were also employed for additional comparison.

**Results:**

The sensitivity of the SmCTF-RDT compared to standard parasitological methods was 100% while the specificity was 39.5%. It was found that the sera from RDT “false-positive” children showed significantly higher antibody titres in IgM-cercarial antigen preparation (CAP) and IgM-soluble egg antigen (SEA) ELISA assays than children identified by parasitology as “true-negatives”.

**Conclusions:**

Although further evaluations are necessary using more accurate reference standard tests, these results indicate that the RDT could be a useful tool for the rapid prevalence-mapping of both *S. mansoni* and *S. haematobium* in schistosome-endemic areas. It is affordable, user-friendly and allows for diagnosis of both schistosome species at the POC.

## Background

Schistosomiasis is a chronic disease caused by infection with parasitic worms of the genus *Schistosoma*. It is endemic in over 70 tropical and sub-tropical countries, with over 200 million people infected and a further 600 million people thought to be at risk. 90% of the people infected reside in Sub-Saharan Africa, where *S. mansoni* and *S. haematobium* infections are prevalent [1].

After the London Declaration of 30^th^ January 2012 momentum towards the control of neglected tropical diseases (NTDs), including schistosomiasis, by 2020 increased markedly [2-4]. Efforts to control morbidity of the disease are being stepped up worldwide and elimination is now an aim following the 2012 WHA Resolution 65.21 which advocates elimination of schistosomiasis in some countries [4,5]. Preventive chemotherapy, or mass drug administration (MDA), using praziquantel is the mainstay of control.

Prior to implementation of MDA treatment strategies, the prevalence of infection in a given area is estimated using the Kato-Katz faecal smear for *S. mansoni* infections, and urine filtration, and/or questionnaires for visible haematuria for *S. haematobium* infections [6]. A control strategy for each area can then be devised following the WHO Guidelines for schistosome control, which stratify control measures based on infection prevalence [6]. While these traditional parasitological methods of diagnosis are often useful in areas of high endemicity, they are less sensitive for diagnosis in people carrying low infection levels [7-13]. In areas of low infection intensity, which will occur if MDA campaigns are successful, more sensitive methods of diagnosis will be needed to ensure implementation of the most appropriate treatment strategy for the given population. This is particularly important for monitoring infection levels and thus part of WHA 65.21 calls for strengthened surveillance, which will require diagnostic methods which can detect low infections. Furthermore, the WHO has recently recommended that preschool-aged children be included in treatment programmes [14]. A significant proportion of these children harbour light infections [15,16], and are therefore more likely to be missed by traditional parasitological methods.

Much effort has been expended in development of more accurate methods for diagnosing schistosomiasis and these include development of molecular methods for detection of parasite specific DNA in urine [17,18]. Detection of *Dra*1 tandem repeats using PCR has been shown to be a promising technique for the diagnosis of urogenital schistosomiasis [13,18]. It may currently be the most sensitive and specific method available for the diagnosis of schistosomiasis in low-endemic areas. However, it is unlikely to become useful for mapping purposes in the near future due to the costs and difficulties associated with performing PCR in the field.

Antibody-detection assays have the merit of high sensitivity [7]. In areas of low infection intensity, sensitivity has previously been shown to decline [19], but despite this they remain one of the best available methods for diagnosis in these areas [20-22]. They are likely to become increasingly useful in Africa as control programmes get underway and infection intensities decline [7,9]. Such has been the case in the People’s Republic of China since the 1980s [23,24]. Antibody-detection methods are often criticised for their lack of specificity but the lack of an accurate ‘gold’ standard diagnostic test makes this parameter difficult to assess. Antibody-positive, egg-negative patients may in fact have infections that are missed by insensitive parasitological methods.

While sensitive and specific diagnostic tests based on antibody-detection are available, those that also meet other ASSURED criteria (Affordable, Sensitive, Specific, User-friendly, Rapid, Equipment-free, Deliverable) are the most likely to be useful in schistosome-endemic areas of low-middle income countries [25]. Most antibody-detection assays currently available are relatively expensive, trained technicians are required to perform the tests, electricity is often needed (e.g. for blood centrifugation) and it can take many hours to obtain results. A test that is affordable, rapid, easy-to-use and works on whole blood will obviate the need for a laboratory or even electricity and allow diagnosis at the point-of-care (POC). POC diagnosis is beneficial not only for individual patients but it will also allow for more rapid mapping of disease prevalence [26] and the acceleration of schistosomiasis control programmes, factors which are important in efforts to meet the 2020 goals [27].

Commercially available POC tests for schistosomiasis include reagent strips for detection of micro-haematuria (e.g. Hemastix®, Bayer; Combur 10 Test® strips, Roche; Medi test combi 9, Macherey-Nagel) and the POC-circulating cathodic antigen (CCA) test (Rapid Medical Diagnostics, Pretoria, South Africa). These have been shown to be useful for diagnosis of *S. haematobium*[28-30] and *S. mansoni* infections [31-36] respectively. Both tests have their advantages and disadvantages: while haematuria reagent strips are very inexpensive at ~ US$0.33/test [28], their sensitivity and specificity are not sufficiently high to make the assay useful for accurate diagnosis at the level of the individual. Haematuria can also occur for other reasons such as inflammation from bacterial cystitis, sexually-transmitted infections, genital mutilation and menstruation [37]. The POC-CCA test is sensitive for *S. mansoni* infections [30-35] but less so for *S. haematobium*[38,39], and its widespread use may be limited to some extent by its cost [20], currently around US$1.75/test [31].

In a collaborative study between the University of Nottingham (Nottingham, UK), BioGlab Ltd. (Nottingham, UK) and Vision Biotech (Cape Town, South Africa) a new rapid diagnostic test (RDT) for POC diagnosis of schistosomiasis has been developed. The test employs *S. mansoni* cercarial transformation fluid (SmCTF) for the detection of anti-schistosome antibodies in human blood. This antigen has been shown to perform equivalently to schistosome soluble egg antigens (SEA) in enzyme-linked immunosorbent assays (ELISA) [40-42]. The SEA-ELISA is regularly employed in travellers’ medicine clinics, and has also been shown to be useful in schistosome-endemic areas [22,24,39,43,44]. The SmCTF-RDT is designed to detect both anti-*S. mansoni* and anti-*S. haematobium* antibodies. Once commercially available it is thought that the SmCTF-RDT will cost approximately US$1/test [45]. A recent evaluation of the RDT for diagnosis of schistosomiasis in preschool-aged children in Cote d’Ivoire suggests that it is at least as sensitive as two Kato-Katz slides for the diagnosis of *S. mansoni* infections and one urine filtration for the diagnosis of *S. haematobium* infections [45]. Another study showed that the sensitivity of the test was however reduced in children aged below three years on the shorelines of Lake Albert, Uganda [46]. Here we further evaluated the diagnostic accuracy of the SmCTF-RDT by comparison to traditional parasitological methods of diagnosis in children of Murehwa, in Mashonaland East province, Zimbabwe, an area co-endemic for both S*. haematobium* and *S. mansoni* infections. ELISAs incorporating *S. haematobium* antigen preparations were also performed to further strengthen the results.

## Methods

### Ethical approval and consent

Institutional and ethical approval was received from the University of Zimbabwe’s Ethical Review Board and the Medical Research Council of Zimbabwe, respectively. In addition permission to work in the indicated area was given by the Provincial Medical Director, by the District Educational Officer and Heads of Schools. Only compliant participants were recruited and they were free to drop out at any point during the study. At the beginning of the study, participants and parents/guardians had the aims and procedures of the project explained fully in the local language, Shona, prior to obtaining consent and assent. All subjects, or parents/guardians of minors, provided informed written consent and assent before parasitology and blood samples were obtained. After collection of all samples, all participants were offered anti-helminthic treatment with the recommended dose of praziquantel (40 mg/kg of body weight) which was administered to all compliant participants by the local physician.

### Study population

This study was part of a larger on-going investigation into the health benefits of repeated treatment of schistosome infections in preschool children. The study population was chosen based on Ministry of health national survey data obtained in 2010 and the study was performed in February 2012 [47,48]. Data from the National survey indicated that overall prevalence in selected schools was >50% for *S. haematobium* and therefore classified as having high endemicity based on WHO criteria [49]. Data of the National survey also indicated a lower prevalence of *S. mansoni* (<20%) and the absence of soil-transmitted helminths (STHs). Testing for both *S. mansoni* and STHs was done using both the Kato-Katz technique and formol-ether concentration. Prevalence of *S. haematobium* in the selected area was checked two weeks before the actual survey by screening children in grade 7 (12-14 years of age). The prevalence within grade 7 was >70% confirming the high prevalence in this area. The study was performed in two villages, Chingwaru and Chingono (31°63’E; 17°52′S and 31°66′E; 17°55′S), Murehwa district, Mashonaland East province in northeast Zimbabwe. The two villages have insufficient safe water and sanitation facilities as confirmed by questionnaire. The villagers are mainly subsistence farmers and have frequent contact with schistosome-infected water. Questionnaire studies showed that infants and preschool children have passive water contact mainly when they accompany older siblings/parents/guardians to the water e.g. being washed in infested water collected from rivers. Older children are exposed to infection while playing, bathing and performing domestic chores. At the time of this study the area had not been included in any schistosome control programmes and participants had not received any prior anti-helminthic treatment.

### Inclusion criteria

To be included in the study participants had to meet following general criteria: 1) should not have received anti-helminthic treatment prior to the study (both assessed by questionnaires to the children or their parents/guardians), 2) be healthy as assessed by a clinical examination conducted by the local nurses. Children were excluded from the final analysis if they had no egg count results for both *S. mansoni* and *S. haematobium*, and also if an egg count result was available for only one species and it was negative, since in such instances the presence of the other species could not be excluded. A total of 97 children met these criteria and were included in the study. 91 children were included in the final analysis (see STARD flowchart: Supplementary information).

### Diagnostic methods

#### Parasitology

For the analysis of *S. haematobium* and *S. mansoni* children submitted up to four urine and four stool samples (over four consecutive days). 10 ml of each urine sample received was processed on the day of collection by a filtration method [50]. Stool samples were prepared and examined on the day of collection using the Kato-Katz faecal smear for detection of *S. mansoni* eggs [51]. A single slide for microscopic examination was prepared from each stool (41.7 mg) and urine sample.

#### Blood collection and analysis

Up to 10 ml of venous blood was collected from study participants (depending on age) by trained nurses and collected in silica-coated tubes (BD Biosciences) without anticoagulant. Blood was stored overnight at 4°C and sera separated by centrifugation at 1800 rpm for 10 min. 10 μl of serum was taken for testing on SmCTF-RDTs, and the rest was initially frozen at -20°C and afterwards stored at -80°C for testing in ELISA and other unrelated studies. Samples were transported to Edinburgh frozen at -80°C prior to immunological assays.

#### SmCTF-RDTs

**Figure 1:**
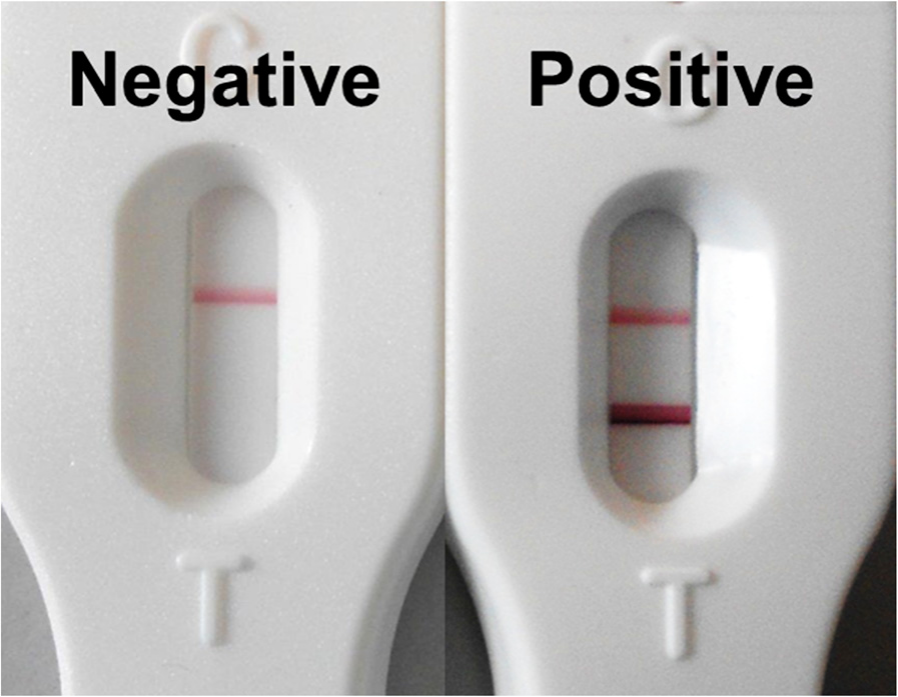
**Negative and positive results on the *Schistosoma mansoni *cercarial transformation fluid rapid diagnostic test (SmCTF-RDT).** The negative test shows the presence of only one band – the control, whereas the positive test shows the presence of a second band. This band shows that there has been a reaction between antibodies in the sample and SmCTF.

SmCTF was produced by BioGlab Ltd. (Nottingham, UK) according to a previously described method [40]. The freeze-dried SmCTF was sent to Vision Biotech (Cape Town, South Africa) for incorporation into the RDTs, which are designed to detect IgG against the SmCTF. Quality control procedures, which involved testing SmCTF-RDTs with schistosome-positive reference sera and European negative controls, were performed prior to distribution of SmCTF-RDTs. In the field, tests were carried out according to the manufacturer’s instructions; briefly, 10 μl of serum was added to a test cassette, followed by 2-3 drops of buffer (provided in the test pack). After 15 min the results were recorded as either positive or negative, depending on whether or not a coloured test band had appeared on the cassette (Figure 1). Read-outs from the test were clear, with positives and negatives easily distinguishable. Readers of the test were blinded with respect to the results of parasitological testing.

#### Serological antibody assays

Sera from the majority of the children included in this study were analysed by ELISA using a *S. haematobium* cercarial antigen preparation (CAP), soluble egg antigens (SEA) and a soluble worm antigen preparation (SWAP). *S. haematobium* antigen preparations were obtained from the Theodor Bilharz Institute (Giza, Egypt). IgM responses against CAP (n = 75) and SEA (n = 75) were recorded and the SWAP-IgG was also measured (n = 77). Sufficient volumes of serum for testing by ELISA were not available from all children, as the amount of blood obtained from young children was very limited in some cases.

ELISAs were performed as previously described [52]. Microtiter plates were coated overnight at 4°C with 5 μg/ml CAP or 5 μg/ml SWAP or with 10 μg/ml SEA, all diluted in carbonate-bicarbonate buffer. Plates were washed with PBS/0.03% Tween20 and blocked for two hours using 5% skimmed milk in PBS/0.03% Tween20. Plates with diluted serum samples (1:100) were incubated for two hours at 37°C. Following three wash steps, anti-human horseradish peroxide-conjugated polyclonal antibodies against IgG and IgM (1:1000; antibodies supplied by Dako, Glostrup, Denmark) were added and incubated for one hour at 37°C. ELISA plates were developed using 2,2’-azino-bis-(3-ethylbenzthiazoline-6-sulfonic acid) solution (ABTS; Southern Biotech, Birmingham, USA). Reactions were stopped after 15 min for SEA-IgM and 10 min for CAP-IgM and SWAP-IgG using 25% hydrochloric acid. Absorbance was read at 405 nm using an Emax precision microplate reader (Molecular devices, Sunnyvale, USA).

### Statistical analysis

The sensitivity and specificity of the RDT were calculated using egg-detection as the reference standard for diagnosis of schistosome infections. Specificity, i.e. the percentage of negative individuals correctly identified as such, was calculated using the formula:

$$\begin{array}{ll} \;\text{Specificity} & =\text{number}\;\text{of}\;\text{true}\;\text{negatives}\;\left(\text{TN}\right)\\ & \;/\left(\text{number}\;\text{of}\;\text{TN}+\text{number}\;\text{of}\;\text{false}\;\text{positives}\;\left(\text{FP}\right)\right). \end{array}$$

Sensitivity, i.e. the percentage of positive individuals correctly identified as such, was calculated using the formula:

$$\begin{array}{ll} \;\text{Sensitivity} & =\text{number}\;\text{of}\;\text{true}\;\text{positives}\;\left(\text{TP}\right)\\ & \;/\left(\text{number}\;\text{of}\;\text{TP}+\text{number}\;\text{of}\;\text{false}\;\text{negatives}\;\left(\text{FN}\right)\right). \end{array}$$

Also calculated were the positive predictive value (PPV), i.e. the proportion of positive test results that are truly positive, and negative predictive value (NPV), i.e. the proportion of negative test results that are truly negative. These were calculated using the formulas:

$$\mathrm{PPV}=\mathrm{TP}/\left(\mathrm{TP}+\mathrm{FP}\right)$$

$$\mathrm{NPV}=\mathrm{TN}/\left(\mathrm{TN}+\mathrm{FN}\right).$$

Statistical analyses were conducted using Stata version 12 (StataCorp. 2011. *Stata Statistical Software: Release 12*. College Station, TX: StataCorp LP) and GraphPad Prism version 5 (GraphPad Software, Inc., La Jolla, USA). Univariate logistic regression analysis was used to determine whether the number of samples of excreta (categorical, x) submitted for analysis had an effect on the outcome of the SmCTF-RDT result (binary, y). Due to small sample sizes, differences in test performance (categorical data) between age groups were analysed using Fisher’s exact test. Antibody data did not meet assumptions allowing for a parametric test. Thus, differences in antibody levels were analysed using the non-parametric Kruskal-Wallis test with multiple comparison by Dunn’s post-test. Statistical significance was assigned at *P* < 0.05 for all analyses. The *P*-value was two-tailed.

## Results

The 91 children included in the final analyses submitted varying numbers of urine and faecal samples over the four days of sampling (see Additional file 1: STARD flowchart in online supplementary information). Five children were missing *S. mansoni* egg count data, but were still included in the analysis since they were positive for *S. haematobium* eggs. The remaining children submitted at least one urine sample and one faecal sample for analysis by filtration for *S. haematobium* eggs and a Kato-Katz slide for *S. mansoni* eggs respectively. The mean age was 7.65 years (range 1-12 years) and there was a 1.4:1 male:female sex ratio. These and other details of the study participants are shown in Table 1.

**Table 1:** Description of the study population

**Age group (years)**	**Sample size (n)**	**Mean age (range)**	**Sex M/F**	***S. haematobium***		***S. mansoni***	
				**Mean egg count ± SEM (range)**	**Prevalence in % (CI)**	**Mean egg count ± SEM (range)**	**Prevalence in % (CI)**
<6	18	4.24 (1-5)	10/8	6.26 ± 2.90 (0-41)	38.9 (17.3-64.3)	0.03 ± 0.03 (0-0.5)	5.6 (0.1-27.3)
6-12	73	8.49 (6-12)	43/30	24.23 ± 7.98 (0-533.0)	56.2 (44.1-67.8)	0.23 ± 0.11 (0-5.5)	12.3 (5.8-22.1)
Total 1-12	91	7.65 (1-12)	53/38	20.67 ± 6.46 (0-533.0)	52.8 (42.0-63.3)	0.19 ± 0.09 (0-5.5)	11.0 (5.4-19.3)

91 children from two villages Chingwaru and Chingono (31°63’E; 17°52′S and 31°66′E; 17°55′S), Murehwa district, Mashonaland East province in northeast Zimbabwe.

SEM - Standard error of mean, CI - Confidence Interval.

The available parasitological data confirmed that five children were infected with *S. mansoni* only, five children had both *S. mansoni* and *S. haematobium* infections, and 43 children were infected with *S. haematobium* only. 38 children were negative for eggs of both schistosome species. The overall prevalence of schistosomiasis was 58.2% (95% confidence interval (CI): 47.4-68.5) with *S. mansoni* prevalence being 11.0% (95% CI: 5.4-19.3) and that of *S. haematobium* being 52.8% (95% CI: 42.0-63.3). Of the *S. haematobium* egg-positive cases, 38 children had light infections (defined by the WHO as <50 eggs per 10 ml urine [49]) and 10 children had heavy infections (defined by the WHO as ≥50 eggs per 10 ml urine). The mean egg count of *S. haematobium* egg-positive children was 39.2 eggs per 10 ml urine (standard error of the mean - SEM ± 11.67). Of the 10 *S. mansoni* egg-positive cases there were two children with moderate infection intensities (as defined by the WHO as 100-399 eggs per gram (epg) [49]) while the remaining 8 had light infections (as defined by the WHO as 1-99 epg). The mean epg of egg-positives was 38.4 (SEM ± 13.68).

The SmCTF-RDT estimated prevalence of anti-schistosome antibodies to be 83.5% (95% CI: 74.5-89.9). The diagnostic accuracy of the RDT compared to the egg-detection methods used as a reference standard is shown in Table 2. All 53 schistosome infections detected by parasitology were also detected by the SmCTF-RDT, and therefore sensitivity was 100% (95% CI: 93.3-100). Of the 38 children that were negative by parasitological methods, only 15 were also negative by the SmCTF-RDT, and therefore specificity was 39.5% (95% CI: 24.0-56.6). The positive predictive value (PPV) and negative predictive value (NPV) were 69.7% (95% CI: 58.1-79.8) and 100% (95% CI: 78.2-100) respectively (Table 2). There was no significant difference between the specificity of the SmCTF-RDT in preschool-aged children (<6 years of age) compared to school-aged children (6-12 years of age) (60.0% and 36.4% respectively; *P* = 0.365) as determined by Fisher’s exact test. There was also no significant difference between the PPV of the SmCTF-RDT in preschool-aged children (<6 years of age) compared to school-aged children (6-12 years of age) (61.5% and 71.4% respectively, *P* = 0.813). Logistic regression analysis showed there was no clear association between the SmCTF-RDT results and the number of urine and faecal samples submitted for examination (odds ratio 0.95, 95% CI 0.57-1.59, *P* > 0.05). Excluding those children who submitted less than two urine and two faecal samples (n = 18) did not alter the outcome of calculations of diagnostic accuracy.

**Table 2:** Diagnostic accuracy of the rapid antibody test for schistosomiasis compared to parasitological techniques as the reference standard

**SmCTF-RDT result**	**Egg-positive children (n = 53)**	**Egg-negative children (n = 38)**	**Sensitivity (95% CI)**	**Specificity (95% CI)**	**PPV (95% CI)**	**NPV (95% CI)**
Positive	53	23	100% (93.3-100)	39.5% (24.0-56.6)	69.7% (58.1-79.8)	100% (78.2-100)
Negative	0	15	-	-	-	-

Accuracy of the *Schistosoma mansoni* cercarial transformation fluid rapid diagnostic test (SmCTF-RDT) for diagnosis of *S. mansoni* and *S. haematobium* infections compared to standard parasitological methods (the Kato-Katz faecal smear and urine filtration respectively).

CI – Confidence Interval; PPV – Positive Predictive Value; NPV – Negative Predictive Value.

**Figure 2:**
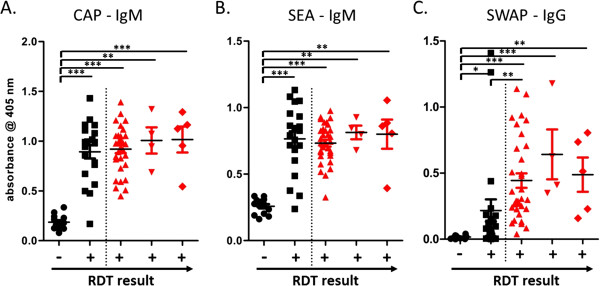
**Antibody isotype levels against different *Schistosoma haematobium *antigen preparations.** Specific antibody isotype levels of the children included in this study from Murehwa, Mashonaland East province, Zimbabwe, against different *Schistosoma haematobium* antigen preparations: **A**: IgM against cercarial antigen preparation (CAP), **B**: IgM against soluble egg antigens (SEA), **C**: IgG against soluble worm antigen preparation (SWAP). Enzyme-linked immunosorbent assay (ELISA) optical density readings are classified according to *S. mansoni* cercarial transformation fluid rapid diagnostic test (SmCTF-RDT) results and infection status as determined by egg counts. (black circle) Egg-negative, (black square) Egg-negative, (red triangle) *S. haematobium* egg-positive, (inverted red triangle) *S. mansoni* and *S. haematobium* egg-positive, (red diamond) *S. mansoni* egg-positive. * *P* < 0.05, ** *P* < 0.01, ***P < 0.001.

For most of the 91 children enrolled in the study sera were available which were further analysed using ELISA with *S. haematobium* CAP, SEA and SWAP. The IgM responses to CAP and SEA in ELISA are presented in Figure 2A and 2B respectively, categorised according to absence or presence of schistosome eggs and SmCTF-RDT results. Both the mean anti-CAP and anti-SEA OD readings of SmCTF-RDT negative results were significantly lower than the mean OD readings of both SmCTF-RDT “true-positives” and SmCTF-RDT “false-positives” (Figure 2A and 2B). There was no significant difference between the mean OD readings of SmCTF-RDT “true-positives” and SmCTF-RDT “false-positives” using either assay. The IgG response to SWAP across the different parasitological findings and RDT results is shown in Figure 2C. The mean anti-SWAP OD readings of SmCTF-RDT negative results were significantly lower than the mean OD readings of both SmCTF-RDT “true-positives” and SmCTF-RDT “false-positives”. The mean OD for SmCTF-RDT “false-positives” was also however significantly lower than that of the RDT-positive/*S. haematobium* egg-positive patients.

## Discussion

Before the onset of schistosomiasis control programmes the prevalence of infection in a given area needs to be estimated in order for implementation of an appropriate MDA strategy. In order to achieve the WHO’s 2020 goals and WHA Resolution 65.21, control efforts will need to be scaled up and accelerated [27]. As we move towards elimination, the prevalence and intensity of schistosome infections will decrease and it will therefore be increasingly difficult to detect these infections using egg detection methods. The need for a more rapid and accurate method of diagnosis is therefore greater than ever.

This study employed a newly-developed RDT that employs SmCTF, an antigen preparation from *S. mansoni* cercariae, for detection of anti-schistosome IgG antibodies in human serum or whole blood (e.g. from a finger-prick [45]). The rapidity with which the test yields a result enables treatment to be administered almost immediately.

In terms of diagnostic accuracy, it has previously been shown that the RDT is as sensitive as two Kato-Katz slides and two POC-CCA tests for the diagnosis of *S. mansoni* infections, and as sensitive as one urine filtration for the diagnosis of *S. haematobium* infections in preschool-aged children of Côte d’Ivoire [45]. While the SmCTF-RDT was highly sensitive for the diagnosis of *S. mansoni* infections in 3-5 year olds of Lake Albert, Uganda, sensitivity was reduced in children aged under 3 years [46]. Evaluation of the SmCTF-RDT has been extended here. The accuracy of the SmCTF-RDT for diagnosis of *S. mansoni* and *S. haematobium* infections was assessed in young children (12 years and under) from Murehwa, located in the Mashonaland East province of Zimbabwe. Those children diagnosed as positive by parasitology were mainly harbouring light infections. The SmCTF-RDT was 100% sensitive compared to parasitological methods for the diagnosis of both schistosomiasis mansoni and haematobium. Specificity was 39.5%, PPV was 69.7% and NPV was 100%, with no difference found in the performance of the test between those children of preschool-aged (<6 years) and those of school-age (6-12 years). The high NPV observed here indicates that the SmCTF-RDT is unlikely to miss any egg-positive infections, and it could be therefore a useful tool for screening purposes. The PPV indicates that 69.7% of the SmCTF-RDT positive results will be “true”, i.e. egg-positive, infections.

The low specificity of the RDT compared to egg-detection methods is consistent with that reported previously [45,46]. Antibody-detection methods are known to give much higher estimates of prevalence than parasitological methods [11,39,53,54] and it has previously been suggested that this may be a direct consequence of the relative insensitivity of the latter [8,9]. In this study up to four Kato-Katz slides and four urine filtrations were performed per child and the prevalence of schistosome infections was as expected from the data of the National survey. It has however been shown previously that infections can still be missed after larger numbers of parasitological examinations [11,55]. Here we therefore further analysed serum samples collected using IgM-ELISA against *S. haematobium* CAP and SEA, and IgG-ELISA against *S. haematobium* SWAP. For the CAP-IgM and SEA-IgM assays, there was no significant difference found between the mean antibody titres of SmCTF-RDT “true-positives” and “false-positives”. Importantly, there was however a significant difference between the mean ODs of SmCTF-RDT-negative/egg-negative children and SmCTF-RDT positive children (irrespective of whether or not eggs were found in excreta). This was not the case with the IgG-ELISA; although there was a significant difference between the mean antibody titres of SmCTF-RDT-negative/egg-negative children and SmCTF-RDT positive children (irrespective of whether or not eggs were found in excreta), there was also a significant difference between the mean ODs of SmCTF-RDT “true-positives” and “false-positives”. SEA and CAP have both previously been shown to be more reactive against anti-schistosome antisera in ELISA than SWAP [56,57]. In this study, the results of the SWAP-IgG ELISA seem to be more comparable to egg counts, which has also been demonstrated previously by Osada *et al.*[58]. The ability of the SmCTF-RDT to detect antibodies as accurately as the ELISAs incorporating SEA and CAP is a promising result. At the pre-treatment stage, the apparent lack of specificity of these tests could mainly be due to the relative insensitivity of parasitological techniques. Anti-schistosome antibodies that are detected by SmCTF are likely induced by immunogens derived from eggs lodged in the tissues of infected humans and it is therefore improbable that SmCTF detects single-sex worm-alone infections [41]. Since schistosomes are long-lived and the studied population here was very young, the likelihood of aborted infections is very small.

### Limitations of the study

Further evaluations are needed in areas where schistosomiasis is non-endemic to determine whether there is cross-reactivity with antibodies against other infections. SmCTF has previously been shown to exhibit some cross-reactivity with sera from other infections in an ELISA format [41], and this cross-reactivity may be responsible to some degree for the apparent lack of specificity of the SmCTF-RDT. Here the studied population was however deemed to be uninfected with STHs based on data from the National survey. More sensitive assays (such as antibody-detection techniques) could be employed in future studies to confirm this to be true.

The sample size used here was limited due to a lack of resources and needs to be increased in future studies. Evaluations into the test’s diagnostic accuracy should now be extended using larger sample sizes, more diverse age groups and in areas of lower endemicity. More accurate reference standard tests, such as an increased number of parasitological examinations and the *Dra* 1 PCR technique [18], should also be employed for more realistic estimates of both sensitivity and specificity.

A sensitive diagnostic tool that can also distinguish between past and present infections will be most useful for monitoring and evaluating control programmes with the ultimate goal of elimination [9]. Antibody-detection tests are often criticised for lacking this ability. The failure of antibody levels to decline rapidly after treatment may however be due in part to sub-curative effects of praziquantel treatment with remaining residual infections after treatment causing persistence of anti-schistosome antibodies [59]. In some treated subjects antibody levels do decline within a period of weeks or months and antibody levels may therefore be indicative of true parasitological cure [60]. Future studies should be focussed towards assessing the diagnostic accuracy of the RDT at the post-treatment stage.

## Conclusions

This study demonstrates that the SmCTF-RDT could be a useful tool for mapping anti-schistosome antibody prevalence prior to implementation of MDA campaigns. It is as sensitive as traditional parasitological methods, and as specific as other routine antibody-detection methods for the diagnosis of both *S. haematobium* and *S. mansoni* infections in preschool-aged and school-aged children of Zimbabwe.

## Competing interests

MJD is the owner of BioGlab Ltd.

## Authors’ contributions

NN carried out the fieldwork and immunoassays, performed the statistical analysis and drafted the manuscript. EMD performed the statistical analysis and drafted the manuscript. FM, NM and TM carried out fieldwork. FM conceived the study, and participated in its design and coordination and helped to draft the manuscript. MJD conceived of the study, contributed materials and helped to draft the manuscript. All authors read and approved the final manuscript.

## Pre-publication history

The pre-publication history for this paper can be accessed here:

http://www.biomedcentral.com/1471-2334/14/165/prepub

## Supplementary Material

Additional file 1 **STARD flowchart.** Standards for Reporting of Diagnostic Accuracy (STARD) flowchart detailing the number of children enrolled in the study and those excluded from the final analyses due to lack of egg count data. Click here for file
