# Supplementary figures and images for: Field evaluation of a new antibody-based diagnostic for Schistosoma haematobium and S. mansoni at the point-of-care in northeast Zimbabwe
Source: BMC Infect Dis. 2014 Mar 26;14:165. doi: 10.1186/1471-2334-14-165 (PMC4021455; doi:10.1186/1471-2334-14-165)

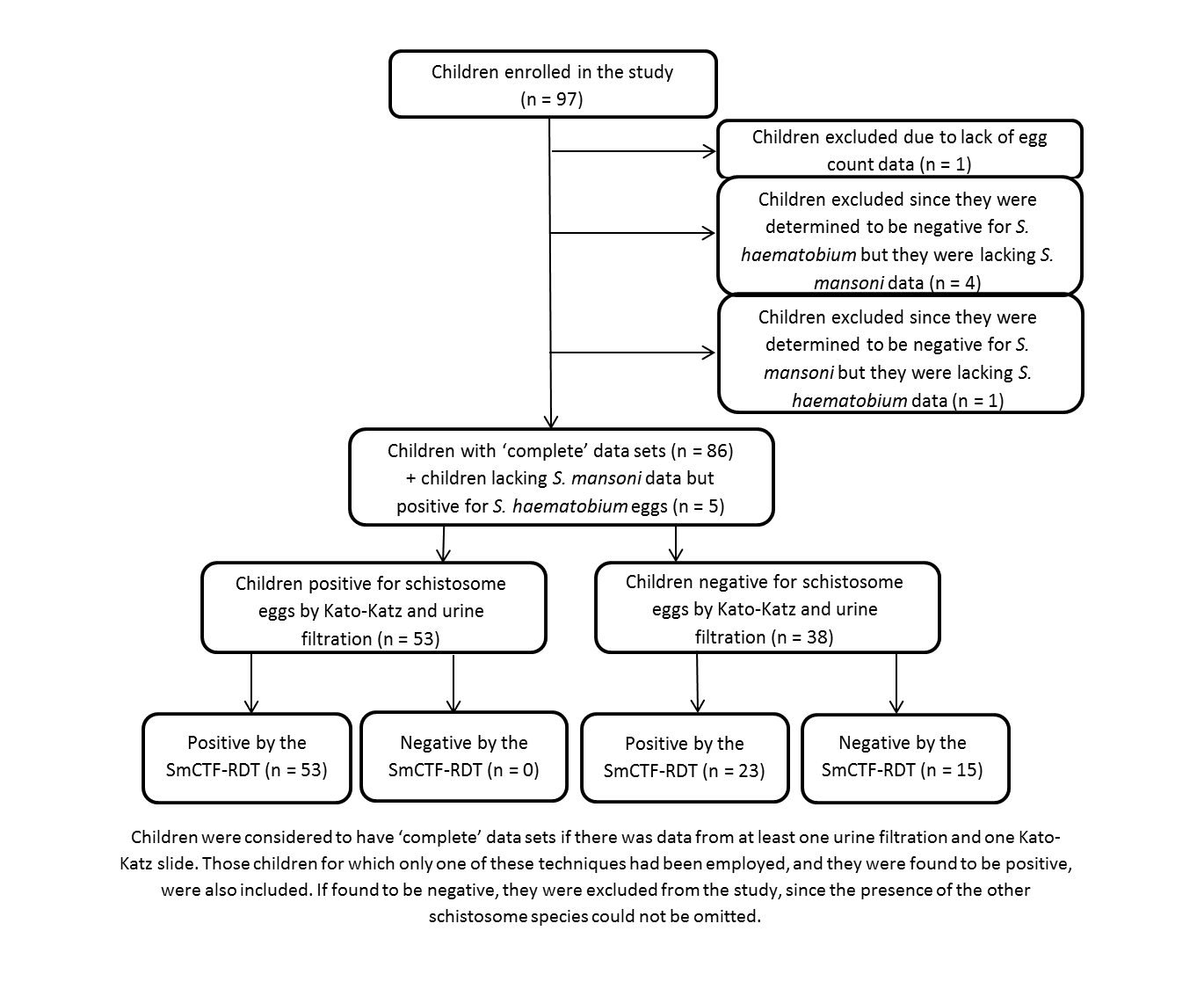

Supplement: Additional file 1 — STARD flowchart. Standards for Reporting of Diagnostic Accuracy (STARD) flowchart detailing the number of children enrolled in the study and those excluded from the final analyses due to lack of egg count data. [file 1471-2334-14-165-S1.tiff]
